# Merkel Cell Polyomavirus DNA in Respiratory Specimens from Children and Adults

**DOI:** 10.3201/eid1503.081067

**Published:** 2009-03

**Authors:** Seweryn Bialasiewicz, Stephen B. Lambert, David M. Whiley, Michael D. Nissen, Theo P. Sloots

**Affiliations:** Sir Albert Sakzewski Virus Research Centre, Brisbane, Queensland, Australia (S. Bialasiewicz, S.B. Lambert, D.M. Whiley, M.D. Nissen, T.P. Sloots); University of Queensland Clinical Medical Virology Center, Brisbane (S. Bialasiewicz, S.B. Lambert, D.M. Whiley, M.D. Nissen, T.P. Sloots); Pathology Queensland; Brisbane (M.D. Nissen, T.P. Sloots)

**Keywords:** Merkel cell, carcinoma, polyomavirus, novel, respiratory, transmission, pediatric, real time PCR, dispatch

## Abstract

Merkel cell polyomavirus (MCPyV) DNA was detected in 7 (1.3%) of 526 respiratory tract samples from patients in Australia with upper or lower respiratory tract symptoms. Partial T antigen and major capsid protein sequences of MCPyV identified in respiratory secretions showed high homology (99%–100%) to those found in Merkel cell carcinoma.

In the past 2 years, several previously unknown human polyomaviruses (PyVs) have been identified: KI virus (KIPyV) (*1*) and WU virus (WUPyV) (*2*) from respiratory samples, and more recently, Merkel cell polyomavirus (MCPyV), most commonly from Merkel cell carcinoma tissue (*3*). MCPyV has been found in integrated and episomal states (*3*); however, a mode of transmission for MCPyV has not yet been proposed. In this study, we sought to identify MCPyV in respiratory specimens by using real-time PCR.

## The Study

This study was conducted exclusively at the Sir Albert Sakzewski Virus Research Centre, Royal Children’s Hospital and Health Service, Brisbane, Queensland, Australia. The necessary ethical approval for this study was obtained from the Ethics Committee of the Royal Children’s Hospital.

Specimens tested in this study (n = 526) were collected from January through December 2003 from 418 pediatric patients (birth to 14 years of age) (n = 450; age range 3 days–13.5 years; mean 1.7 years; median 0.8 years) and 71 adult patients (n = 76; age range 14.3 years–80.1 years; mean 47.1 years; median 52.5 years) who were hospitalized or sought treatment at hospitals in Queensland, Australia, for upper or lower respiratory tract symptoms. Most (95.6%) samples were nasopharyngeal aspirates (NPAs); the remainder were bronchoalveolar lavage specimens, bronchial washing specimens, and endotracheal aspirates. These samples were collected as part of a previous study that tested for influenza viruses A and B, adenoviruses, human metapneumovirus, respiratory syncytial virus, and parainfluenza viruses 1, 2, and 3, in addition to KIPyV and WUPyV (*4*).

Samples were screened by using an MCPyV real-time PCR that was specific for the VP2/3 region. Briefly, 10 pmol of each primer MCPyV-2.0–4367F (5′-GGCAGCATCCCGGCTTA-3′) and MCPyV-2.0–4399R (5′-CCAAAAAGAAAAGCATCATCCA-3′) and 4 pmol of dual-labeled probe MCPyV-2.0–4371-Prb (5′-FAM-ATACATTGCCTTTTGGGTGTTTT-BHQ1-3′) in a 25-μL reaction using QIAGEN Quantitect Probe master mix (QIAGEN, Doncaster, Victoria, Australia) with 2 μL of template were run on a LightCycler 480 (Roche Diagnostics, Castle Hill, New South Wales, Australia) under the following conditions: incubation at 95°C for 15 min, followed by 45 cycles of 95°C for 15 s and 60°C for 1 min. Quantitative real-time PCR was not performed because of limited applicability due to inherent variability in respiratory sample collection. The presence of MCPyV in samples positive by real-time PCR was then confirmed by using partial large T antigen (LT3) and major capsid protein (VP1) conventional MCPyV detection PCR assays of Feng et al. (*3*). All PCRs were performed in a unidirectional workflow through dedicated suites for reagent preparation, PCR setup, and amplification. Ten random real-time PCR–negative samples, 10 water control samples, and template-negative control samples were used to exclude amplicon or sample cross-contamination. A clinical sample that was positive for MCPvV by all 3 assays and confirmed by sequencing was used as a positive control for all PCRs.

Thirty-one (5.9%) samples produced positive results in the real-time PCR screening. Of these, 8 (1.5%) could be confirmed by only 1 conventional PCR, and 7 (1.3%) yielded positive results in all 3 MCPyV PCRs. All positive detections were in NPA samples. This variation in detection rates could have been due to the unforeseen nonspecificity of the real-time PCR, or to the inherent lower sensitivity of conventional PCRs, because most real-time PCR–positive samples produced late signals at cycle threshold values ≈40. We chose a conservative algorithm, in which a sample must have been positive in all 3 assays to be considered a true positive. This rule may have led to an underestimation of MCPyV prevalence in this sample population.

All MCPyV-positive samples were collected during the spring and early summer months (October–December 2003). Five (1.1%) of these samples originated from pediatric patients, which suggests early acquisition of virus. In fact, all 4 MCPyV-positive patients who were not immunosuppressed were <2 years of age (Table 1). This age range coincides with ages of those who experience primary infections of many other human viruses (*5*,*6*). Of the 3 immunosupressed MCPyV-positive patients, 2 were adults (2.6%), and 1 was a 6.6-year-old child. Further information on each MCPyV-positive patient is listed in Table 1.

**Table 1:** Detection of MCPyV in nasopharyngeal aspirates in persons with respiratory signs and symptoms, Queensland, Australia, 2003*†

Sample	Ct	Codetections	Patient characteristics				
			Age	Sex	Immune status	Clinical history	Signs and symptoms
MCV-B1 (LT3: FJ009185, VP1: FJ009188)	29.0	–	52.0 y	M	Suppressed	Acute myeloid leukemia	Flu-like
MCV-B2 (LT3: FJ009186, VP1: FJ009189)	33.0	–	6.6 y	F	Suppressed	Heart transplant, T-cell lymphoma	Coryza, fever, sore throat, ear pain
MCV-B3 (LT3: FJ009187, VP1: FJ009190)	31.5	–	47.7 y	M	Suppressed	Heart transplant	–
MCV-B4	36.9	–	8 mo, 8 d	M	Competent	–	Bronchiolitis
MCV-B5	37.5	–	1 mo, 26 d	M	Competent	–	Rhinorrhea, cough
MCV-B6	37.4	–	9 mo, 27 d	F	Competent	–	
MCV-B7	38.9	Adenovirus, WUPyV	1.6 y	F	Competent	–	Fever, convulsions

*MCPyV, Merkel cell polyomavirus; Ct, cycle threshold; WUPyV, WU polyomavirus. †Clinical histories and symptoms were noted when available. Associated GenBank accession numbers are shown in parentheses.

We could not discern whether the higher frequency of detections in adults was representative of the general adult population or whether the detections were overrepresented due to a small sample size or the immune status of the adult cohort. The integration status of the detected MCPyV DNA was not able to be ascertained; however, for 1 patient for whom multiple samples were tested, MCPyV was neither detected 2 months before nor 3 months after MCPyV-B4 was detected, which suggests that, at least in this particular patient, MCPyV may have had transitory presence or activity. Multiple samples were not available for any of the other MCPyV-positive patients.

Three MCPyV (MCV-B1/B2/B3)–positive samples were further investigated through bidirectional sequencing of their LT3 (FJ009185, FJ009186, and FJ009187, respectively) and VP1 (FJ009188, FJ009189, and FJ009190, respectively) assay amplification products. The sequences of the 3 samples showed high homology to those of the 2 previously described MCPyV strain sequences, MCC350 (EU375803) and MCC339 (EU375804) (*3*); similarities in the LT3 and VP1 target regions ranged from 99.6%–100.0% and 99.0%–99.6%, respectively (Table 2).

**Table 2:** Nucleotide variation in LT3 and VP1 sequences from 3 MCPyV strains detected in respiratory secretions from patients, Queensland, Australia, 2993*†

Isolate name	Nucleotide position and change					
	LT3		VP1			
	843		3825	3875	3909	4022
**MCC350‡ (EU375803)**	**G**		**T**	**C**	**C**	**T**
MCC339‡ (EU375804)	C		C	G	T	A
MCV-B1 (LT3: FJ009185, VP1: FJ009188)	C		C	–	–	–
MCV-B2 (LT3: FJ009186, VP1: FJ009189)	C		C	G	–	A
MCV-B3 (LT3: FJ009187, VP1: FJ009190)	C		C	–	–	–

*LT3, partial large T antigen; VP1, major capsid protein; MCPyV, Merkel cell polyomavirus. †Nucleotide changes are in relation to the prototype strain (**boldface**). Dashes indicate homology with MCC350. Nucleotide positions are given in a 5′ → 3′ early to late orientation, respective to alignment with MCC350. GenBank accession numbers are shown in parentheses. ‡Original MCPyV strain sequences described by Feng et al. (*3*).

Clinical notes were available for 5 of the 7 MCPyV-positive patients and indicated that these patients exhibited a variety of upper and lower respiratory tract symptoms. MCPyV was the sole viral agent detected in 6 of the 7 samples (Table 1). However, due to limited remaining volumes, study samples were not screened for other known respiratory pathogens, including coronaviruses and rhinoviruses, which may have led to a higher codetection rate, similar to that for KIPyV (*1*,*4*) and WUPyV (*2*,*4*).

## Conclusions

To date, MCPyV has been most commonly studied and detected in the context of Merkel cell carcinoma (*3*,*7*), but it has also been identified in Kaposi sarcoma tissue (*3*), primary squamous cell carcinoma tissue (*7*), keratoacanthoma tissue from an organ recipient (*8*), in normal sun-exposed skin (*7*), and in a small number of control tissues (normal skin, small bowel, hemorrhoids, appendix, and gall bladder) (*3*). In this study, MCPyV was detected in 1.3% of respiratory samples collected from symptomatic persons. This description of the presence of MCPyV in the respiratory tract raises questions about the transmission and respiratory pathogenicity of this newly described PyV.

Analogous to MCPyV, both KIPyV (*1*,*4*) and WUPyV (*2*,*4*) have been found globally in respiratory specimens with a high prevalence of codetected viruses, but clear evidence for a causal association with respiratory illness has yet to be identified. These PyVs may be merely transmitted through the respiratory route or detected during periods of reactivation (*9*). Similarly, JC and BK PyVs are suspected of being transmitted by inhalation and are occasionally detected in respiratory samples, yet patients generally remain asymptomatic or exhibit nonspecific upper respiratory tract symptoms (*10*,*11*). Like these other PyVs, MCPyV may potentially be transmitted through the respiratory route and become latent in other sites, such as epidermal tissue, by systemic spread, in a similar fashion to murine PyV (*12*). Clearly, further studies using larger sample populations, expanded respiratory pathogen detection methods, and control groups are needed to elucidate what role, if any, MCPyV plays in upper and lower respiratory tract illness.

On the basis of the ages and immune status of the MCPyV-positive patients, we can hypothesize that MCPyV may have an infectious cycle similar to that of other human PyVs, in which the virus is acquired early in life, undergoes a period of latency, and then becomes reactivated in the event of immunosuppression (*10*). Sequence data from positive specimens indicate that MCPyV found in respiratory secretions is similar to the viruses identified within Merkel cell carcinomas. Whether and how the virus is translated from the respiratory tract to skin cells, if it is able to be transported systemically, and its role in the malignant transformation of Merkel cells are all questions worthy of further investigation.
